# Spinal motor outputs during step-to-step transitions of diverse human gaits

**DOI:** 10.3389/fnhum.2014.00305

**Published:** 2014-05-15

**Authors:** Valentina La Scaleia, Yuri P. Ivanenko, Karl E. Zelik, Francesco Lacquaniti

**Affiliations:** ^1^Department of Systems Medicine, University of Rome Tor VergataRome, Italy; ^2^Centre of Space Bio-medicine, University of Rome Tor VergataRome, Italy; ^3^Laboratory of Neuromotor Physiology, Santa Lucia FoundationRome, Italy

**Keywords:** EMG, motoneurons, spinal mapping, tiptoe, uphill, backward, walking

## Abstract

Aspects of human motor control can be inferred from the coordination of muscles during movement. For instance, by combining multimuscle electromyographic (EMG) recordings with human neuroanatomy, it is possible to estimate alpha-motoneuron (MN) pool activations along the spinal cord. It has previously been shown that the spinal motor output fluctuates with the body's center-of-mass motion, with bursts of activity around foot-strike and foot lift-off during walking. However, it is not known whether these MN bursts are generalizable to other ambulation tasks, nor is it clear if the spatial locus of the activity (along the rostrocaudal axis of the spinal cord) is fixed or variable. Here we sought to address these questions by investigating the spatiotemporal characteristics of the spinal motor output during various tasks: walking forward, backward, tiptoe and uphill. We reconstructed spinal maps from 26 leg muscle EMGs, including some intrinsic foot muscles. We discovered that the various walking tasks shared qualitative similarities in their temporal spinal activation profiles, exhibiting peaks around foot-strike and foot-lift. However, we also observed differences in the segmental level and intensity of spinal activations, particularly following foot-strike. For example, forward level-ground walking exhibited a mean motor output roughly 2 times lower than the other gaits. Finally, we found that the reconstruction of the spinal motor output from multimuscle EMG recordings was relatively insensitive to the subset of muscles analyzed. In summary, our results suggested temporal similarities, but spatial differences in the segmental spinal motor outputs during the step-to-step transitions of disparate walking behaviors.

## Introduction

Muscle activity during human locomotion is coordinated by tens of thousands of alpha-motoneurons (MNs), organized along the spinal cord (Romanes, 1951; Sharrard, 1964; Tomlinson and Irving, 1977). Bio-imaging techniques are being developed to increase our understanding of this spinal neural function, but generally these techniques (e.g., functional magnetic resonance imaging) remain difficult or impossible to use for studying the spinal cord during walking (Harel and Strittmatter, 2008; Stroman et al., 2014). Furthermore, most available techniques do not distinguish between activation of sensory neurons and that of MNs in the spinal cord. However, mapping muscle activations onto the rostrocaudal location of MN-pools in the human spinal cord provides a compact representation of the total motor output (Yakovenko et al., 2002; Ivanenko et al., 2006; O'Donovan et al., 2008; Monaco et al., 2010; Warp et al., 2012). This mapping also provides a complementary perspective to conventional approaches to understanding neural control, which often rely on detailed analyses of individual muscle activity and inter-muscular coordination (e.g., D'Avella and Bizzi, 2005; Ting, 2007; Giszter et al., 2010; D'Avella et al., 2013).

In previous studies, the spinal mapping method was used to investigate development and aging (Monaco et al., 2010; Ivanenko et al., 2013b), as well as the relationship between the spatiotemporal organization of the spinal motor output and the biomechanics of human locomotion (Ivanenko et al., 2008; Cappellini et al., 2010; MacLellan et al., 2012). In particular, Cappellini et al. (2010) found that, during both forward and backward walking on level ground, the spatial activity of the spinal cord fluctuated with the center-of-body-mass (COM) motion, with bursts of activity around touchdown and foot lift-off. However, it is not known whether these bursts of activity around touchdown and toe-off are generalizable to other gaits nor is it clear if the spatial location of the activity (along the rostrocaudal axis of the spinal cord) is fixed or variable for different gaits. A better understanding of spinal motor outputs during different locomotion modes may provide further insights into adaptability and modularity of neural control (Lacquaniti et al., 2013; Bagnall and McLean, 2014), interspecies comparison (Carlson-Kuhta et al., 1998; Yakovenko et al., 2002), and may thus also have important clinical implications (Grasso et al., 2004; Scivoletto et al., 2007; Coscia et al., 2011; Oetgen and Peden, 2012; Hoogkamer et al., 2014).

Thus, the purpose of this study was to investigate these questions about motor output during several different locomotor tasks: forward, backward and digitigrade (tiptoe) walking on level ground, and walking on an inclined surface. These tasks may also be relevant to clinical, rehabilitation or sport applications. For instance, toe walking is observed in patients with various neurologic and developmental abnormalities (Oetgen and Peden, 2012), backward locomotion is used increasingly in sports and rehabilitation (Hoogkamer et al., 2014) and uphill walking may be appropriate exercise for obese individuals at risk for musculoskeletal pathology or pain (Haight et al., 2014). We used the recordings from 26 leg muscles (including intrinsic foot muscles that have not typically been considered) to reconstruct spinal motor outputs with specific interest in identifying common and idiosyncratic features across locomotor gaits.

## Materials and methods

### Experimental protocol

We recorded surface electromyograms (EMGs) and foot motion for 8 subjects (4 males, 4 females, 25.6 ± 2.6 years old, 1.78 ± 0.11 m, 76 ± 16 kg) during 4 ambulation tasks: walking forward, backward, tiptoe and uphill (20% inclined grade), all at 4 km/hr. These tasks were selected to represent biomechanically distinct walking gaits that were cyclic (for EMG analysis purposes) and could be performed at fixed speed on a treadmill. The treadmill speed was selected because it was sufficiently fast to distinguish myoelectric activity from the baseline noise (at slower speeds some muscle EMGs were small and therefore difficult to quantify), but also slow enough that most subjects could perform all the tasks. However, two out of the eight subjects were not able to walk backward at 4 km/hr. Each walking trial lasted 40 s and was performed barefoot on a standard treadmill. Prior to data collection, the subjects were trained on each task, allowing them time to acclimate to the various walking conditions, and all subjects gave informed consent prior to participation. The protocol was approved by the Ethics Committee of the Santa Lucia Institute.

We also collected several additional trials to help identify maximum contraction (MC) magnitude for each muscle EMG. Before collecting the walking data, we asked subjects to perform a set of quasi-static maneuvers against manual resistance. These included: flexing/extending/abducting the toes, plantarflexing/dorsiflexing/inverting/everting the ankle, flexing/extending the knee, flexing/extending/abducting/adducting the hip and flexing the back. Each exercise was performed for 5 s, during which subjects were instructed to perform maximal contractions. Two additional forward walking trials (3 and 5 km/hr) were also recorded and used to help determine MC values.

### Data collection

We recorded kinematic data bilaterally at 100 Hz using a Vicon-612 system (Vicon, Oxford, UK) with nine cameras. Infrared reflective markers (diameter 14 mm) were place bilaterally over the following landmarks: greater trochanter (GT), lateral femural epicondyle (LE), lateral malleolus (LM), heel (HE), and fifth metatarsophalangeal joint (5MP).

**Figure 1:**
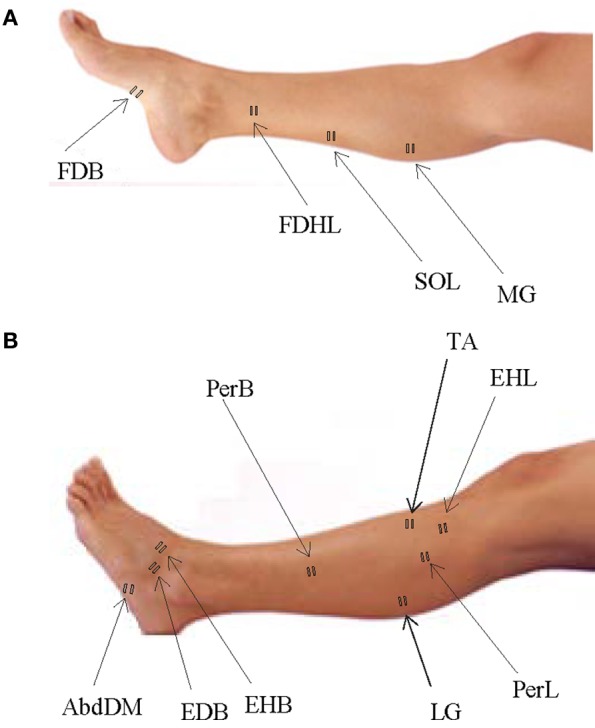
**Sites of EMG electrode placement for muscles in the foot and shank segments. (A)** Plantar surface of the foot and medial aspect of the leg with sites of electrode placement. **(B)** Dorsal surface of the foot and lateral aspect of the leg with sites of electrode placement.

We recorded EMG activity by means of surface electrodes from 26 muscles simultaneously on the right side of each subject. These included one muscle from the lower back (erector spinae (ES) at L2 level), two muscles from the buttocks [gluteus maximus (Gmax) and gluteus medius (Gmed)], 11 muscles from the thigh [iliopsoas (Ilio), tensor fasciae latae (TFL), sartorius (Sart), adductor magnus (AddM), adductor longus (AddL), vastus medialis (Vmed), vastus lateralis (Vlat), rectus femoris (RF), biceps femoris long head (BFL), biceps femoris short head (BFS), semitendinosus (Semit)], six muscles from the shank [tibialis anterior (TA), peroneus longus (PerL), peroneus brevis (PerS), medial gastrocnemius (MG), lateral gastrocnemius (LG), soleus (Sol)], and six muscles from the foot [extensor hallucis longus (EHL), flexor digitorum/hallucis longus (FDHL), extensor hallucis brevis (EHB), extensor digitorum brevis (EDB), abductor digit minimi (AbdDM), flexor digitorum brevis (FDB)]. The activations of flexor digitorum longus and flexor hallucis longus were indistinguishable in our surface EMG recordings, due to close proximity of the muscles, and thus are reported together. We placed EMG electrodes based on suggestions from SENIAM (seniam.org), the European project on surface EMG. To this end, we located the muscle bellies by means of palpation and oriented the electrodes along the main direction of the fibers (Winter, 1991; Kendall et al., 2005). The placement of EMG electrode for muscles in the foot and shank segments is illustrated in Figure 1 for convenience since some of the foot muscles are less commonly recorded in literature.

All EMGs were recorded at 4000 Hz using a Delsys Trigno Wireless System (Boston, MA), except the flexor digitorum brevis which was recorded using a synchronized Delsys Bagnoli System (at 1000 Hz). Due to the recording site of flexor digitorum brevis (on the plantar surface of the foot), the lower-profile Bagnoli electrode was needed. Some electrodes became partially or fully detached during testing, and signals were thus not usable. These EMGs were removed on a subject-specific basis. On average we analyzed 23.4 ± 1.7 muscle EMG from each subject.

### Data processing

**Figure 2:**
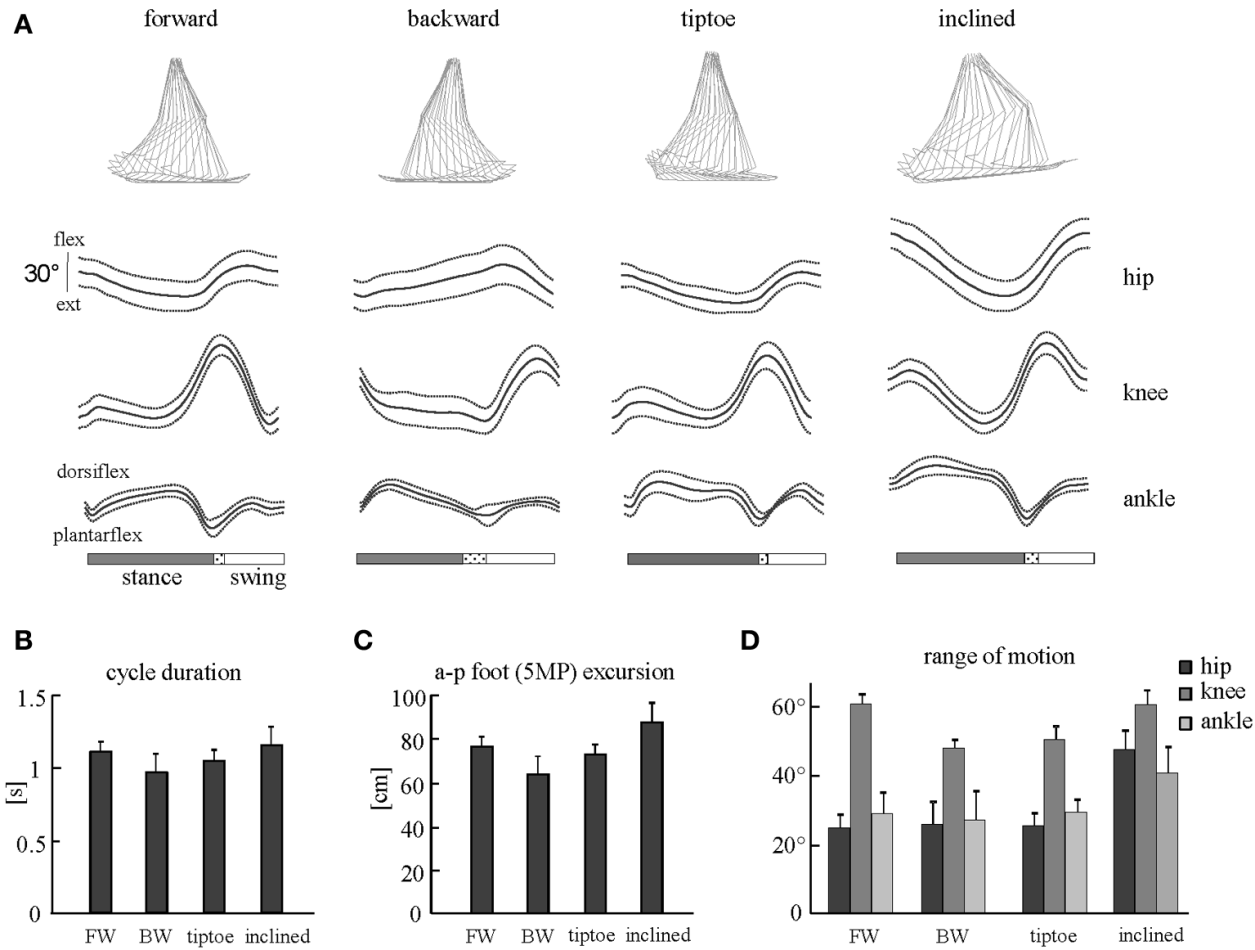
**Kinematic patterns during forward, backward, tiptoe and uphill (20% inclined) walking at 4 km/hr. (A)** Ensemble averages (±SD) of hip, knee, and ankle joint angles of the right leg. Hip and knee angles increase in flexion, ankle angle in dorsi-flexion. The dotted region between stance and swing phases depicts inter-subject standard deviation (SD), and is centered at average foot-lift. *On the top*: stick diagrams for a single stride in one representative subject. **(B)** Cycle duration (mean +SD) for different gaits. **(C)** Anterior-posterior foot (5MP marker) excursion. **(D)** Peak-to-peak amplitudes (+SD) of angular motion.

The beginning of the gait cycle (foot-strike) was defined based on kinematic events. We used vertical height of the right HE marker for forward walking and limb elevation angle (based on maximum GT-5MP virtual segment displacement) for backward, tiptoe and inclined walking. Similarly, for stance to swing transition (foot-lift) we used limb elevation angle (based on minimum GT-5MP virtual segment displacement) for forward, tiptoe and inclined walking, and minimum vertical height of the HE marker for backward walking. The usage of these criteria was based on the different kinematic endpoint (foot) behaviors for the various gaits (Ivanenko et al., 2007). While the differences in definition of gait cycle initiation may have introduced minor time shifts between tasks, all muscle EMGs for a single task shift together in time and so for each individual gait this did not impact the fidelity of spinal map reconstruction. General gait parameters [cycle duration, anterior-posterior foot (5MP) excursion] and joint (ankle, knee and hip) angular range of motion were calculated to characterize the kinematics of gaits studied (Figure 2).

**Figure 3:**
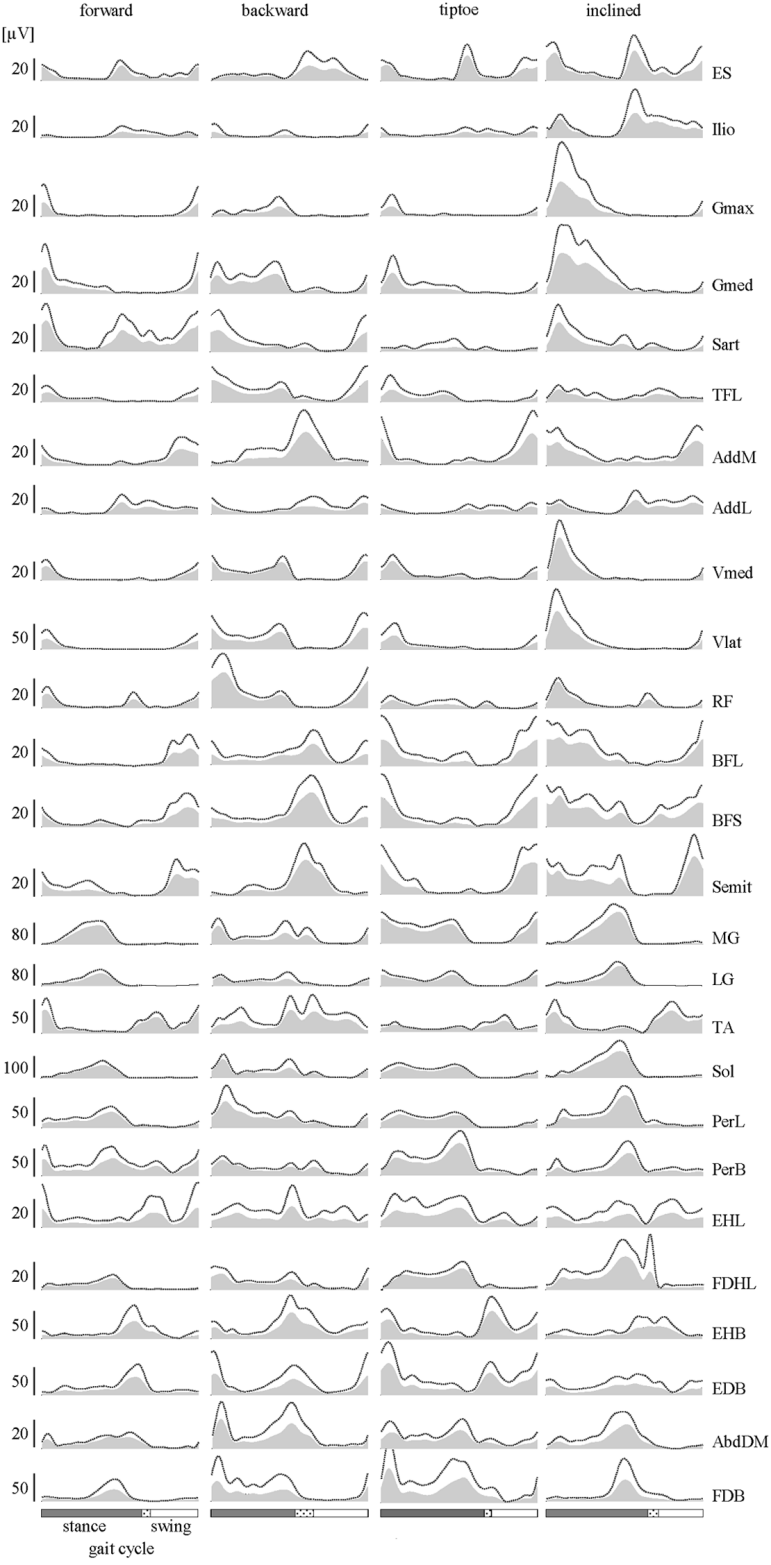
**EMGs. Subject-averaged patterns of muscle activity during forward, backward, tiptoe and uphill (20% inclined) walking at 4 km/hr**. Mean EMGs and inter-subject standard deviations are plotted across a normalized gait cycle from foot-strike to ipsilateral foot-strike. The dotted region between stance and swing phases depicts inter-subject standard deviation (SD), and is centered at average foot-lift.

**Figure 4:**
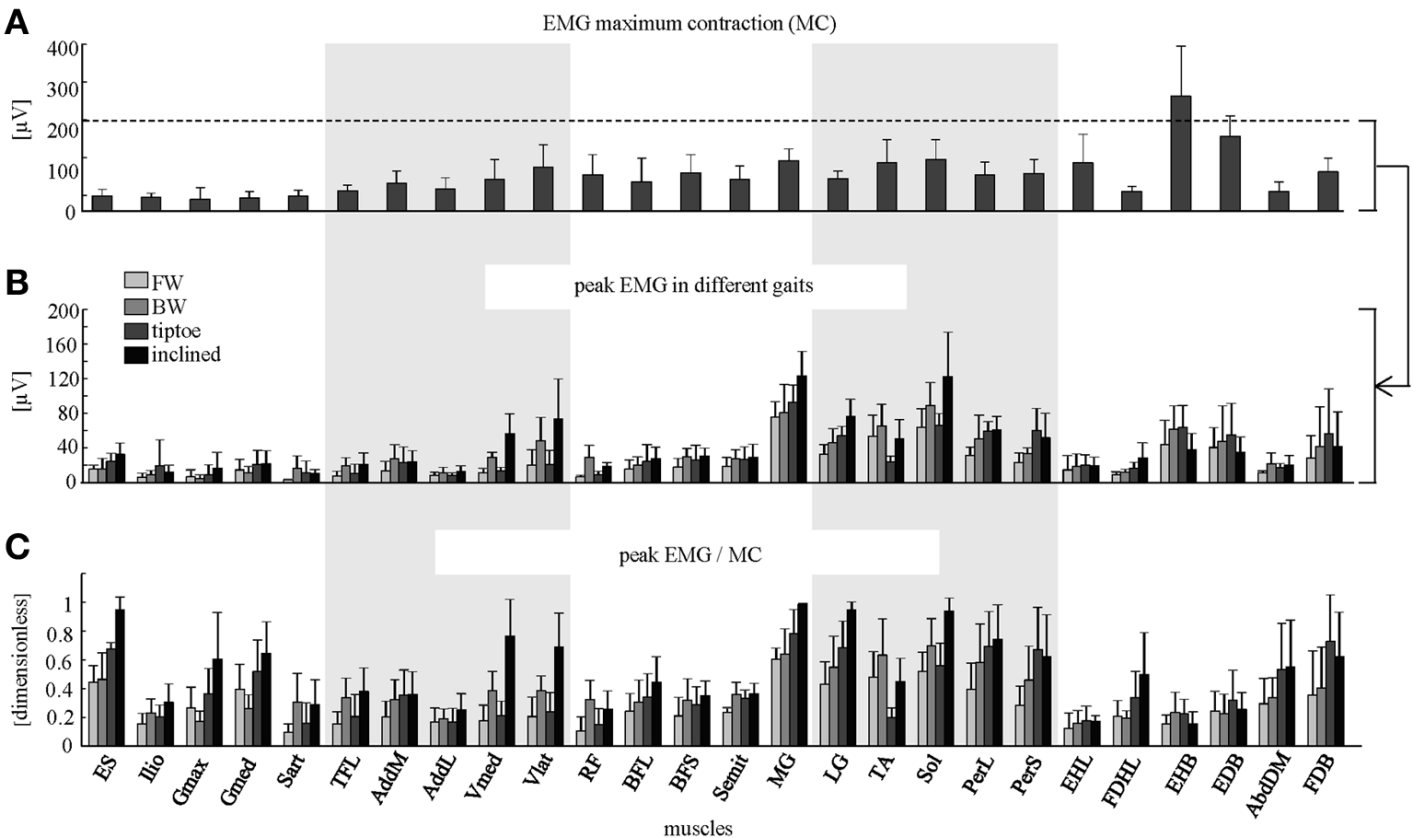
**Peak EMG magnitudes. (A)** Maximum contraction (MC) values (+SD) are shown (based on peak EMGs from dynamic and quasi-static trials; see Methods for full details). Peak EMG amplitudes (+SD) are depicted in μV **(B)** and as a percentage of MC **(C)** for forward, backward, tiptoe and inclined walking at 4 km/hr.

We processed EMG data using standard filtering and rectifying methods. We applied a 30 Hz high-pass filter, then rectified the EMG signals and applied a 10 Hz low-pass filter (all filters, zero-lag 4th order Butterworth). To reduce residual baseline noise, which appear as offsets in the EMG envelopes, we subtracted the minimum signal from each EMG. This assumes that at some point during walking each muscle is effectively “off” (not actively contracting). Some subjects exhibited artifacts in the foot muscles, generally linked to foot-strike and foot-lift events. In order to remove these artifacts, high-pass filtering of these muscles was performed using a 150 Hz cut-off frequency (rather than 30 Hz). A prior study on cut-off frequency (Potvin and Brown, 2004) and informal tests on locomotor EMGs (Zelik et al., 2014) confirmed that this artifact-removal filter had minimal effect on the shape of the muscle activation pattern. For illustrative purposes, the EMGs filtered at higher cut-off frequency were then rescaled to match peak amplitude of the 30 Hz filtered signal (Figures 3, 4). However, this rescaling procedure did not affect the calculation of the motor output since EMGs were eventually normalized to their MC amplitudes before mapping to the spinal cord (detailed below).

We divided EMGs into gait cycles based on foot kinematics, then interpolated each stride to 200 time points, and finally averaged across gait cycles (individually for each subject and task). This yielded an (*m* × 200) EMG matrix for each task, where *m* equaled the number of muscles analyzed. Inter-subject mean (and standard deviation) values for EMG were then computed from these subject-specific data. In addition to calculating the ensemble-averaged EMGs (across strides and subjects), we also present some EMG waveforms of individual strides in order to examine inter-stride variability in the spinal motor output.

### EMG normalization

We normalized EMGs by the MC magnitude across all trials. Normalization was performed to account for the differences in μV magnitudes recorded between muscles. We defined MC magnitude as the muscle's maximum EMG signal from either dynamic (walking) or quasi-static trials (during which subjects were instructed to perform maximal contractions against manual resistance, see *Experimental Protocol*). Thus, all EMGs were considered on a scale from 0 to 1, where 0 indicates that a muscle is inactive and 1 represents maximum muscle activation. Across all quasi-static trials (EMGs were low-pass filtered as described previously), we looked at a sliding 1-s window (by incrementally shifting each time step) and computed the average EMG during each. The highest average EMG found during any 1-s window was defined as the maximum quasi-static activation magnitude. Similarly, maximum dynamic activation magnitude was defined for each muscle as the peak stride-averaged EMG across all walking tasks. The normalization constant for each muscle was then defined as the larger of the quasi-static and dynamic activation magnitudes.

We note that normalization to muscle physiological cross sectional area (PCSA) was not used in this study for reconstructing the segmental spinal outputs, which has occasionally been done in the past (e.g., MacLellan et al., 2012; Ivanenko et al., 2013b). This is because the number of motor units for each muscle is not related to PCSA in a simple way (e.g., number of motor units does not scale proportionally with size of muscle; Feinstein et al., 1955; Christensen, 1959; McComas et al., 1997; McComas, 1998).

### Motor output calculations

To characterize the spinal motor output, EMG-activity was mapped onto the estimated rostrocaudal location of MN-pools in the human spinal cord from L2 and S2 segments. Because this method has been thoroughly documented in previous papers (Ivanenko et al., 2006, 2013b; MacLellan et al., 2012), we describe it only briefly here. The maps were constructed by adding up the contributions of each muscle to the total activity at each spinal segment, using the myotomal charts of Kendall et al. (2005) to link muscles to their spinal innervation levels (see Figure S1). The motor output pattern of each spinal segment *S*_*j*_ was estimated by the following equation:

(1) $$S_{j}=\frac{\sum_{i\;=\;1}^{m_{j}}\left(\frac{k_{ji}}{n_{i}}\times EMG_{i}\right)}{\sum_{i\;=\;1}^{m_{j}}\left(\frac{k_{ji}}{n_{i}}\right)}\times MN_{j}$$

where EMG_i_ represents the normalized, subject-specific envelope of muscle activity, *k*_*ji*_ is a weighting coefficient for the *i*-th muscle (to signify if the *j*-th spinal level is a major, *k*_*ji*_ = 1, or minor, *k*_*ji*_ = 0.5, MN source, see Figure S1), *m*_*j*_ is the number of muscles innervated by the *j*-th spinal segment, and *n*_*i*_ is the total number of spinal levels that innervate the *i*-th muscle, again accounting for major and minor sources (for instance, for the soleus muscle, *n*_*i*_ = 1 + 1 + 0.5 = 2.5, see Figure S1). Thus, the fractional part of Equation 1 can range in value from 0 (inactive) to 1 (maximum activation of that spinal segment). To account for size differences in MN pools at each spinal level, this fractional activity value was then multiplied by the segment-specific number of MNs (*MN*_*j*_). This MN pool size normalization primarily affects the boundary segments L2 and S2, which contain 2–3 times fewer MNs than the other segments (Table S1, Tomlinson and Irving, 1977). We note that Equation 1 is slightly modified with respect to our previous studies (Grasso et al., 2004; Ivanenko et al., 2006, 2013b) in order to better account for the different number of muscles that innervate each spinal segment and the heterogeneity in the MN pools along the lumbosacral enlargement. Thus, our updated calculation yields spinal motor output in units of number of (active) MNs.

The primary assumptions implicit in this analysis are that (1) the rectified EMG provides an indirect measure of the net firing rate of MNs for each muscle (Yakovenko et al., 2002), and (2) the set of recorded muscles is representative of the total motor output from each spinal segment. The first assumption seems reasonable given that mean EMG has been found to increase linearly with the net motor unit firing rate (Hoffer et al., 1987; Day and Hulliger, 2001). However, a limitation is that this method does not account for confounds due to other physiological properties, such as the effects of muscle length or velocity on the EMG signals. To test the second assumption, we compared the activation maps obtained from all 26 recorded muscles with those obtained from reduced subsets of muscles (detailed in *Muscle subset analysis* section below).

To obtain the averaged (across subjects) spinal maps, we calculated the spinal motor output for each subject based on stride-averaged EMGs, and then we averaged it across subjects. We computed two summary metrics to describe the spinal maps: *mean segmental output* and *mean temporal output*. For each condition, we averaged the motor output patterns over the entire gait cycle to find the subject-specific *mean segmental output* and then averaged it across subjects to obtain mean ± SD. Similarly, we averaged the motor output across the spinal segments L2 to S2 to find the *mean temporal output* across the gait cycle. From this mean temporal output waveform, we found the maximum peak in the first half of the stance phase and defined it as activation *burst 1*, and the peak in the second half of the gait cycle as *burst 2*. To characterize the total intensity of the spinal output for each task, we computed for each subject the *mean motor output* by averaging across both spatial segments and gait cycle, and then we averaged it across subjects. In addition to creating subject-specific spinal maps from stride-averaged EMG envelopes, we also computed maps for individual strides and compared them with those obtain from ensemble-averaged strides.

### Muscle subset analysis

Practical considerations limit the number of muscles from which we could record. Thus, there is the potential issue of how the specific selection of the muscles affects the resulting spatiotemporal maps of MN activity. To evaluate the sensitivity of the spinal maps approach we compared the motor outputs obtained from analyzing all 26 muscles with those obtained from subsets of these muscles. Subsets were chosen as follows: (1) the 20 non-foot muscles (TA, Sol, MG, LG, RF, Vmed, Vlat, AddL, AddM, ES, TFL, PerL, PerB, BFL, BFS, Semit, Sart, Ilio, Gmax, and Gmed) and (2) 12 commonly recorded muscles (TA, Sol, MG, LG, RF, Vmed, Vlat, ES, TFL, BFL, Semit, and Gmax). For forward walking we also made 26 additional comparisons by correlating maps from each unique set of 25 muscles (i.e., by systematically eliminating each individual muscle) with the map constructed from all muscles. The correlation coefficient (r) was calculated for each subject and condition. Averaged correlation coefficients were then reported for each comparison.

### Statistics

To compare activation waveforms we computed linear correlations (*r*-values). For instance, to compare segmental activations of individual subjects with those of averaged maps, correlation coefficient was computed for each subject and each segment, and then they were averaged first across subjects for each segment and then across segments. Similarly, to compare the maps obtained by different sets of muscles, correlation coefficient was computed for each segment, and then the data for all segments were averaged. Since correlation coefficients have non-normal distributions, their mean estimates were computed based on the normally distributed, Z-transformed values.

Repeated measures (RM) ANOVA was used to evaluate differences in the kinematics and the *mean motor output* across different gaits, and *post-hoc* Tukey's HSD test was used to determine statistical significance. Since only six out of the eight subjects were able to walk backward at 4 km/hr their missing data for this condition for the ANOVA were replaced by the unweighted mean value estimated from all other subjects. Reported results are considered significant for *p* < 0.05.

## Results

### Kinematics

General gait parameters and ensemble-averaged joint angular movements are reported in Figure 2. We observed that cycle duration and foot excursion were slightly but significantly lower for backward walking than for forward and inclined walking (*p* < 0.006, Figures 2B,C). These two parameters were also larger for inclined walking relative to tip-toe walking (*p* < 0.03). The range of hip and ankle angular motion was significantly larger during inclined walking than for the other tasks (*p* < 0.001, Figure 2D). The peak-to-peak amplitude of the knee joint oscillations was significantly smaller for backward and tip-toe walking than for forward and inclined walking (*p* < 0.0002, Figure 2D).

### EMG

Lower-limb EMGs (Figure 3) were qualitatively consistent with those reported elsewhere in the literature for forward (Winter, 1991; Ivanenko et al., 2006), backward (Thorstensson, 1986; Grasso et al., 1998; Ivanenko et al., 2008), tiptoe (Perry et al., 2003; Romkes and Brunner, 2007) and inclined walking (Lange et al., 1996; Franz and Kram, 2012). In this study we extended the number of recorded muscles relative to our previous studies (Ivanenko et al., 2006, 2013b). In particular, we included intrinsic foot muscles, which demonstrated their own unique activation patterns with bursts principally around the stance to swing transition of gait (Figure 3).

Averaged EMG waveforms for the deeply located and interconnected muscles during forward walking were consistent with those reported in the literature. The deep hip flexors (Ilio) demonstrated the major peak of activity around lift-off (Rab, 1994; Andersson et al., 1997; Ivanenko et al., 2008). EMG recordings of AddL and AddM showed main bursts at foot lift-off and during swing, respectively (Winter, 1991). The activity of BFL and BFS (at the end of swing and beginning of stance) was similar to that reported by University of California Berkeley (1953). Intramuscular recordings of foot muscle activity (Gersten et al., 1956; Mann and Inman, 1964) showed a good correspondence with our data (Figure 3). Specifically, EHL activity showed two peaks around foot lift-off and heel strike, respectively, while the FDHL showed activity beginning in early stance and continuing until the foot lift-off (Gersten et al., 1956). The EDB and AbdDM became active ~20% of the cycle and the FDB at 40% of the cycle, remaining active until just before foot lift-off (Mann and Inman, 1964).

The amplitude of EMG signals (in μV) varied considerably across muscles, both during walking and in terms of MC (Figures 4A,B). We found that normalizing to MC tended to increase the relative activation magnitude of proximal muscles (e.g., ES, Ilio, Gmax, Gmed, TFL, AddL, Sart, Vmed) and some intrinsic foot muscles (e.g., FDHL, AddDM) and thus their contribution to the spinal maps (Figure 4C).

### Average spinal maps

**Figure 5:**
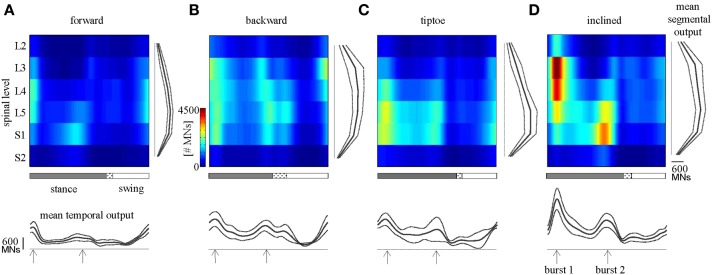
**Spinal maps**. Depicted here are estimates of averaged (across subjects) spatiotemporal spinal motor outputs computed from EMGs for **(A)** forward, **(B)** backward, **(C)** tiptoe, and **(D)** uphill (20% inclined) walking, all at 4 km/hr. Motor output (reported in units of number of MNs) is plotted as a function of gait cycle and spinal segment level. Waveforms plotted below the maps correspond to the mean temporal output pattern averaged first across all 6 segments and then across subjects (mean ± SD, *n* = 8 subjects). Note the tendency for peaks to occur around early and late stance (labeled as burst 1 and 2). Curves to the right of maps represent the mean segmental output averaged first across the entire gait cycle and then across subjects. In the gait cycle, the dotted region between stance and swing phases depicts inter-subject standard deviation (centered at average foot-lift).

**Figure 6:**
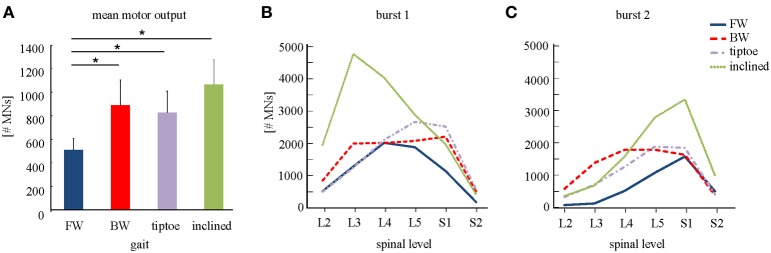
**Spinal motor output. (A)** Depicted are mean (+SD) motor outputs (averaged across both gait cycle and spinal levels). Asterisks denote significant differences between conditions. Segment-specific magnitudes of motor output are also shown for **(B)** burst 1 of spinal activity (occurring after foot-strike; see Figure 5), and **(C)** burst 2 (occurring around foot-lift).

We observed task-specific spinal motor outputs for each walking condition (Figure 5), although with qualitative similarities in temporal profile. In particular, two prominent periods of activity were observed in the mean temporal output of each task (Figure 5, bottom): the first following foot-strike (~5–10% of the gait cycle) and the second preceding foot-lift (~40–55%). However the timing of the second burst relative to foot-lift varied considerably between tasks, occurring later in tiptoe than forward walking, for example (Figure 5). In contrast to the qualitative temporal similarities, we found substantial differences in the spatial localization and intensity of spinal activation for each gait (Figure 5). In particular, we found that the mean motor output (spinal activation averaged across the entire gait cycle and all spinal segments) was significantly lower for forward walking than for the other tasks (*p* < 0.01, Figure 6A). We also found that the loci of mean segmental outputs shifted somewhat as a function of gait (Figure 5, see plots to the right of each spinal map). For instance, forward and tiptoe walking exhibited principle activations in L5 and S1, whereas backward and inclined walking showed a more distributed output with roughly similar intensities from L3 to S1. These differences in spatial level of spinal activation were even more evident during the major “spots” of activity (identified as burst 1 and 2 from the mean temporal output). During burst 1 (after foot-strike, Figure 6B) peak motor output was at the spinal level L3 for inclined walking, L4 for forward walking, L5 for tiptoe walking and S1 for backwards walking (although this gait exhibited a relatively constant intensity from L3-S1). Differences were less evident for burst 2 (Figure 6C), when most gaits exhibited peak motor outputs from spinal segments L5 and S1.

### Subject-specific spinal maps

**Figure 7:**
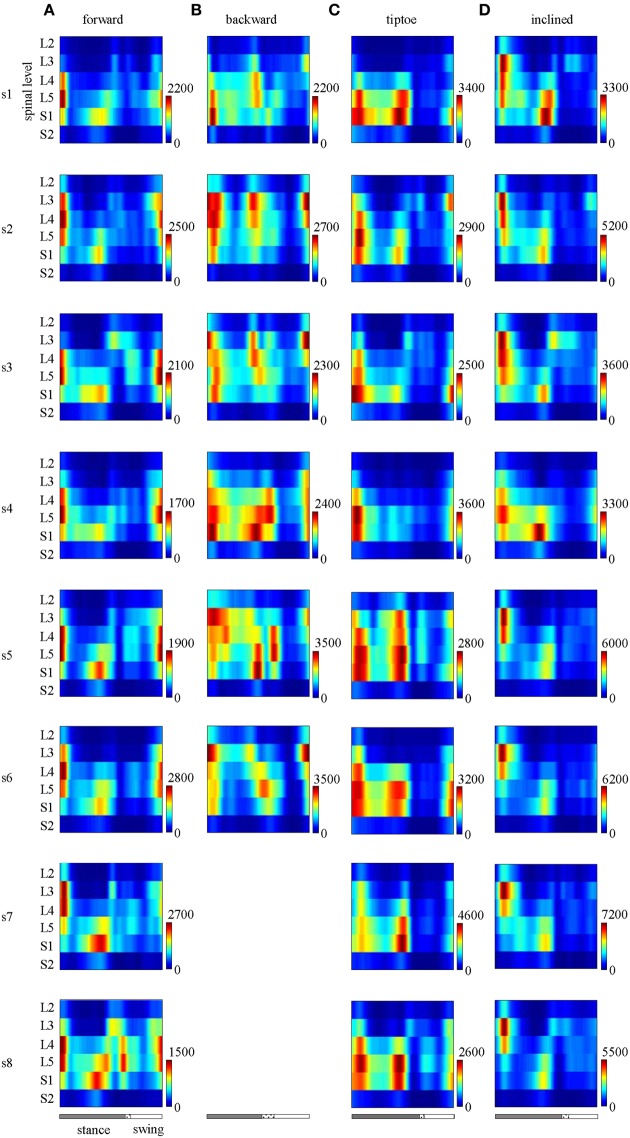
**Spinal maps of MN activity of the lumbosacral enlargement in all subjects for all gaits (A–D)**. Note similar temporal features (main peaks around foot-strike and foot-lift) of the segmental output between individual and averaged (Figure 5) spinal maps.

The major features observed in the average spinal maps were also present in subject-specific maps. In particular, 6 out of the 8 tested subjects exhibited bimodal (two peaked) motor output profiles for all gaits (Figure 7). The remaining 2 subjects also showed the bimodal temporal profile for most gaits except for forward walking (s8 subject, Figure 7A) and backward walking (s5 subject, Figure 7B). In these cases, mean temporal output was found to have an additional peak at the beginning of the swing phase. Individual subjects also exhibited small differences in the segmental level of spinal activation, particularly during load acceptance following foot-strike (Figures 5, 7). Nevertheless we found a strong correlation between subject-specific and average maps (0.85 ± 0.13 depicted in Figure 5), consistent with previously published findings (Ivanenko et al., 2006).

**Figure 8:**
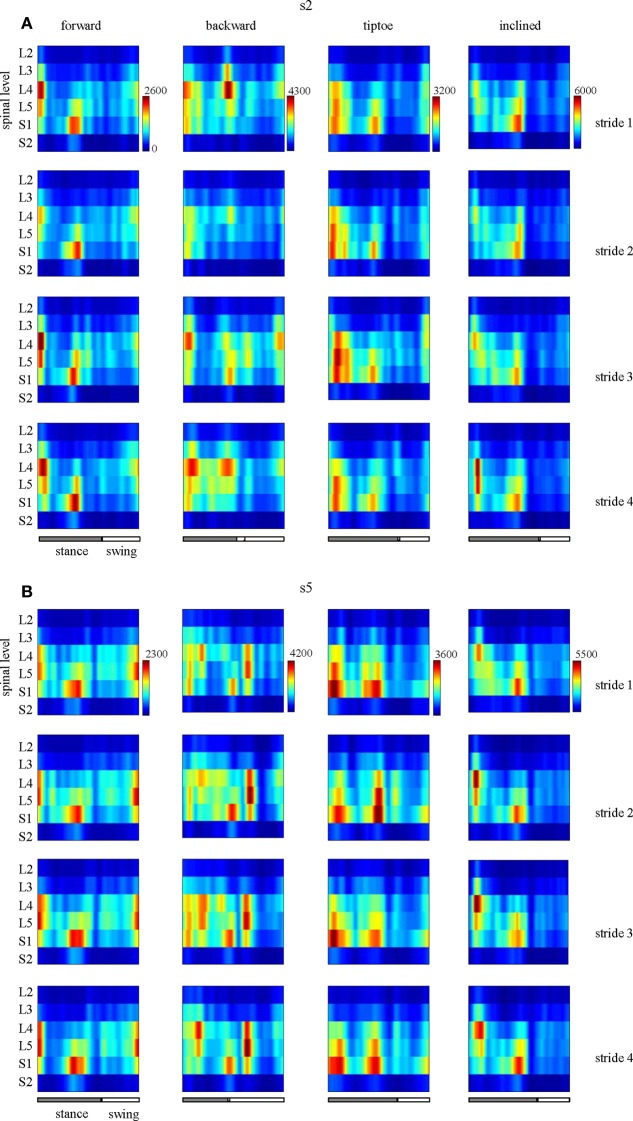
**Examples of spatiotemporal maps of MN activity of the lumbosacral enlargement in two subjects [s2 and s5, (A,B), respectively] for all gaits**. For each individual, four individual strides are shown. Note similar spatiotemporal features (activity around foot-strike and foot-lift) of the segmental output between individual and averaged (Figure 7) strides for these subjects.

EMG profiles exhibit stride-to-stride variability related to dynamic stability and walking speed maintenance (Hausdorff, 2007; Kang and Dingwell, 2009). Examples of the spinal maps of individual strides are illustrated in Figure 8. Despite individual variations in the segmental level of spinal activation, the major features depicted in the stride-averaged maps (Figure 7) are representative of the general trends in individual strides (Figure 8). We found the mean correlation coefficient between segmental output waveforms of individual and ensemble-averaged strides was 0.90 ± 0.04 (average from Table 1).

**Table 1:** **Inter-stride variability in the segmental spinal output for different gaits**.

	**FW**	**BW**	**Tiptoe**	**Inclined**
L2	0.90 ± 0.05	0.85 ± 0.02	0.86 ± 0.06	0.94 ± 0.04
L3	0.90 ± 0.05	0.83 ± 0.02	0.87 ± 0.06	0.94 ± 0.04
L4	0.93 ± 0.01	0.79 ± 0.03	0.89 ± 0.02	0.93 ± 0.03
L5	0.87 ± 0.05	0.72 ± 0.04	0.89 ± 0.01	0.88 ± 0.04
S1	0.91 ± 0.02	0.84 ± 0.03	0.93 ± 0.01	0.92 ± 0.01
S2	0.94 ± 0.02	0.85 ± 0.06	0.93 ± 0.01	0.92 ± 0.02

*For each subject, correlation coefficients between segmental motor outputs (based on 26 recorded muscles) of individual and ensemble-averaged strides were obtained and averaged across strides. Then, for each spinal segment, these correlation coefficients were averaged across subjects and reported in this table (mean ± SD)*.

### Sensitivity to the number of muscles analyzed

We found that the spinal maps were relatively insensitive to the subset of muscles analyzed. Spinal maps computed from 20 and 12 muscle subsets were strongly correlated with the maps computed from the full set of 26 recorded muscles, with average correlation coefficients between 0.98–0.99 and 0.91–0.96, for each task (Table 2). The motor outputs evaluated at each individual spinal segment were also found to be in good agreement, with r values (always greater than 0.9 using 20 muscles, and generally greater than 0.85 using 12 muscles). The only exception was that, with 12 muscles, the L5 segment correlation dropped to 0.74–0.90. Forward walking maps obtained by excluding a single recorded muscle were also highly correlated with those obtained from the full set of 26 muscles (*r* = 0.99 ± 0.01).

**Table 2:** **Sensitivity of spinal maps to the muscle subset analyzed**.

	**20 muscles**				**12 muscles**			
	**FW**	**BW**	**Tiptoe**	**Inclined**	**FW**	**BW**	**Tiptoe**	**Inclined**
L2	1.00	1.00	1.00	1.00	0.94	0.97	0.91	0.98
L3	1.00	1.00	1.00	1.00	0.94	0.96	0.86	0.98
L4	0.99	0.99	0.99	0.99	0.92	0.93	0.91	0.94
L5	0.92	0.97	0.99	0.95	0.74	0.90	0.83	0.74
S1	0.96	0.99	0.99	0.99	0.92	0.98	0.95	0.98
S2	0.99	0.99	0.98	1.00	0.99	0.99	0.99	1.00
Total	0.98 ± 0.03	0.99 ± 0.01	0.99 ± 0.01	0.99 ± 0.02	0.91 ± 0.08	0.96 ± 0.03	0.91 ± 0.05	0.94 ± 0.09

*Correlation coefficients are reported for comparisons between motor outputs based on all 26 recorded muscles and those obtained from subsets of 20 or 12 muscles. For each gait, correlations are reported for individual spinal segments and for the total spinal map (mean ± SD)*.

## Discussion

The overall behavior of the body and limbs during walking is determined by the interplay of neural and mechanical factors. Here we observed that spinal motor outputs corresponded to the major phases of biomechanical force production during diverse walking tasks (Winter, 1991; Perry et al., 2003; DeVita et al., 2007; Franz and Kram, 2012). Specifically, the elevated MN outputs during the gait cycle produce muscle contractions during the step-to-step transition, in which both limbs act to redirect the body's velocity in a way that is thought to improve walking economy (Donelan et al., 2002). However, during the step-to-step transition, we observed differences in the loci of the segmental spinal activity across gaits (Figures 5, 6). This suggests that even if similar biomechanical functions are performed by the limbs (i.e., redirection of the body during the transition), it may be accomplished differently, through a gait-specific coordination of muscles. Thus, high-level features of locomotion may be flexibly encoded by neural circuits to generate muscle activation patterns based on gait-specific constraints and feedback.

Various neural control strategies have been proposed for transforming such task-level goals to muscle-level execution, for example using a hierarchical, modular architecture under feedback control (Ting et al., 2012). The pulse-like features of the spinal motor output observed in this study may be consistent with “drive-pulse” rhythmic elements or neural primitives, which have been hypothesized to underlie the spinal circuitry of animals (Giszter et al., 2007). Although the precise neuronal substrates remain largely unknown (but see Hart and Giszter, 2010), it is believed that a crucial role is played by central pattern generators (Grillner, 1981). Specifically, it has been proposed that motor activation patterns may emerge from a multi-layered organization of the spinal neural networks with two functionally distinct levels, one for rhythm generation and the other for muscle pattern generation (McCrea and Rybak, 2008). In this study we found that the spatial loci of MN pool activations depends greatly on the walking task (Figures 5, 6), indicating that “drive pulse” rhythmic elements may be significantly modulated by task-specific sensory feedback. Since muscle activation timing was linked to major force production events around foot touchdown and foot-lift, it suggests that pre-programmed motoneuronal drive may be principally mediated by afferent force and kinematic-related feedback (Duysens et al., 1998; Nielsen and Sinkjaer, 2002; Pearson, 2004). There is also supporting evidence from a previous study on cats that neuromotor coordination may be modulated by critical points that correspond to key biomechanical events (Saltiel and Rossignol, 2004).

It is worth noting that we observed similarities in the spinal activation maps across subjects (Figure 7) and strides (Figure 8, Table 1), specifically in terms of temporal activation peaks around foot-strike and foot-lift. Thus the spinal mapping methodology seem to provide a robust and repeatable means to reconstruct MN pool activity. Meanwhile, previous literature has demonstrated that the spinal maps do vary for individuals with neuromotor impairments (Grasso et al., 2004; Coscia et al., 2011; Ivanenko et al., 2013a) and throughout the aging process (Monaco et al., 2010) and during childhood development (Ivanenko et al., 2013b). Taken together, the robustness of the methodology and the population-specific activations suggest that spinal mapping approach may be useful for assessing or differentiating gait performance in clinical populations.

It is also interesting to compare spinal maps between plantigrade and digitgrade gaits. Human adults typically walk with a characteristic heel-to-toe progression (plantigrade gait), whereas many animals walk only on their toes (digitgrade gait). In this study we observed roughly a doubling of the intensity of spinal motor output during tiptoe walking (Figure 6A), which is known to incur increased energetic costs compared to plantigrade gait (Cunningham et al., 2010). This increase in motor output was due, in part, to differences in the spinal activity after foot-strike, which was both increased in magnitude and spatially shifted toward more distal segments (L5/S1). The spinal maps for human tiptoe walking were, however, qualitatively different from maps constructed from digitigrade feline locomotion (Yakovenko et al., 2002). In cats, the primary MN activation during walking occurs during midstance and with roughly constant intensity, likely the result of neuromechanical differences associated with their flexed limb posture and quadrupedal gait. This comparison also highlights the potential utility of spinal maps for studying interspecies motor control.

There are several limitations to the spinal mapping approach, many of which have been previously documented (Cappellini et al., 2010). Briefly, the reconstruction and interpretation of spinal maps assume anatomical similarity of motor pools across individuals and that rectified EMG provides an indirect measure of the net MN firing rate (Yakovenko et al., 2002). Another potential concern is related to EMG cross-talk, which is always a potential issue with surface EMG recordings: in particular for deep muscles like Ilio that have a relativity small superficial region for recording and for smaller foot muscles (e.g., flexor digitorum longus) that are in close proximity to larger calf muscles. In the previous study (Ivanenko et al., 2013b), the cross-talk issue was addressed by modeling the potential effect of different levels of cross-talk in the EMG profiles. The spinal segmental output was reconstructed by adding up incrementally the magnitude of cross-talk from adjacent muscles (from 10 to 100%). While the intensity and the width of the main loci of activation could be affected by adding cross-talk, this procedure did not give rise to the appearance of new loci of activation or significant time shifts in the spinal maps. Given the similar spinal mapping methodology in this study, we do not expect that the similarities in spinal maps reconstructions (based on different set of muscles EMGs) were due to cross-talk. Furthermore, the spinal maps during walking have been shown to be similar when reconstructed from EMGs obtained using surface and intramuscular electrodes (Ivanenko et al., 2006). Consistent maps have also been produced in different studies (Ivanenko et al., 2008; Cappellini et al., 2010; Monaco et al., 2010; Coscia et al., 2011; MacLellan et al., 2012). We only tested four walking tasks (Figure 5), but other gaits may show additional (e.g., skipping) or temporally shifted (e.g., running) spots of activity specific for force production in those gaits (Ivanenko et al., 2008). Finally our analysis was also based on a limited set of muscles. However, we found the spinal maps to be relatively robust and insensitive to the subset of muscles analyzed (see Results, Table 2), presumably because the lumbosacral enlargement innervates numerous muscles and each muscle is innervated by several segments.

In summary, we found that the MN activation patterns exhibited two major bursts during diverse walking tasks, one around foot-strike and the other around foot-lift, but with differences in the segmental level and intensity of the spinal activity. We also found further evidence that spinal MN mapping provides a robust method for estimating spinal motor output, which is relatively insensitive to the subset of EMGs analyzed. We also suggest that spinal motor mapping can be used to assess the recruitment of specific motor pools when using epidural electrical stimulation or corticospinal neuroprostheses for restoring locomotor functions (Capogrosso et al., 2013; Borton et al., 2014).

### Conflict of interest statement

The authors declare that the research was conducted in the absence of any commercial or financial relationships that could be construed as a potential conflict of interest.

## Supplementary material

Here we report the anatomical data used to reconstruct the segmental motor output during human locomotion (see Methods). These data include segmental muscle innervation in humans (Figure S1) and the number of MNs in each segment of the human spinal cord (Table S1).

Figure S1 **Spinal segment muscle innervation**. This graphic is adapted from Kendall et al. (2005), which compiled segmental innervation charts for muscles by integrating the anatomical and clinical data of several different sources. The blue color in the chart below denotes an innervation agreed upon by five or more sources, and the light blue color denotes agreement of three to four sources. In our spinal map analysis, we used the weighting coefficient (*k*_*ji*_) to define the major (1) and minor (0.5) innervation segments for each muscle.

Table S1 **Number of motoneurons (MNs) in each segment of the human spinal cord (adapted from Tomlinson and Irving, 1977)**.

## Abbreviations

AbdDM: abductor digiti minimi
AddL: adductor longus
AddM: adductor magnus
BFL: biceps femoris, long head
BFS: biceps femoris, short head
BW: backward walking
EDB: extensor digitorum brevis
EHB: extensor hallucis brevis
EHL: extensor hallucis longus
EMG: electromyography
ES: erector spinae (at L2 level)
FDB: flexor digitorum brevis
FDHL: flexor digitorum/hallucis longus
FW: forward walking
Gmax: gluteus maximus
Gmed: gluteus medius
Ilio: iliopsoas
LG: gastrocnemius lateralis
MC: maximum contraction
MG: gastrocnemius medialis
MN: motoneuron
PerL: peroneus longus
PerS: peroneus brevis
RF: rectus femoris
Sart: sartorius
SD: standard deviation
Semit: semitendinosus
Sol: soleus
TA: tibialis anterior
TFL: tensor fasciae latae
Vlat: vastus lateralis
Vmed: vastus medialis
GT: greater trochanter
LE: lateral femur epicondyle
LM: lateral malleolus
HE: heel
5MP: fifth metatarsophalangeal joint.
