# Supplementary figures and images for: Spinal motor outputs during step-to-step transitions of diverse human gaits
Source: Front Hum Neurosci. 2014 May 15;8:305. doi: 10.3389/fnhum.2014.00305 (PMC4030139; doi:10.3389/fnhum.2014.00305)

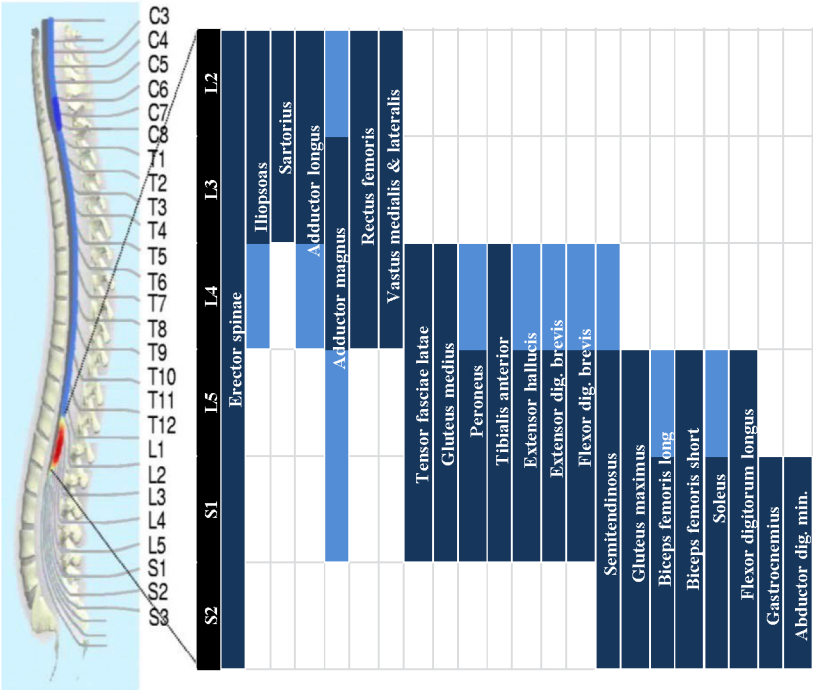

Fig. S1 (Appendix)

Supplement: Figure S1 — Spinal segment muscle innervation. This graphic is adapted from Kendall et al. (2005), which compiled segmental innervation charts for muscles by integrating the anatomical and clinical data of several different sources. The blue color in the chart below denotes an innervation agreed upon by five or more sources, and the light blue color denotes agreement of three to four sources. In our spinal map analysis, we used the weighting coefficient (kji) to define the major (1) and minor (0.5) innervation segments for each muscle. [file Presentation1.PDF]
