# Supplementary material for: Spinal motor outputs during step-to-step transitions of diverse human gaits
Source: Front Hum Neurosci. 2014 May 15;8:305. doi: 10.3389/fnhum.2014.00305 (PMC4030139; doi:10.3389/fnhum.2014.00305)
Supplement: Table S1 — Number of motoneurons (MNs) in each segment of the human spinal cord (adapted from Tomlinson and Irving, 1977). [file DataSheet1.DOCX]

Table S1. Number of motoneurons (MNs) in each segment of the human spinal cord (adapted from Tomlinson and Irving, 1977).

| Spinal Level | MNs |
| --- | --- |
| L1  L2  L3  L4  L5  S1  S2  S3  Total Number | 806  5146  12765  12069  12674  10372  4216  409  58457 |
